# Examining the interplay between media-related professional development, media knowledge, and teacher self-efficacy in STEM and non-STEM domains

**DOI:** 10.3389/fpsyg.2026.1658293

**Published:** 2026-03-19

**Authors:** Kris-Stephen Besa, Annalisa Biehl, Svea Isabel Kleinert, Sabrina Zinner, Dan Verständig

**Affiliations:** 1 Institute for Educational Science, General Didactics and Teaching Research, University of Münster, Münster, Germany; 2 Faculty of Biology, Biology Education Research and Learning Lab, Universitat Duisburg-Essen, Duisburg, Germany; 3 Humanities Section, Empirical Educational Research, Universitat Konstanz, Konstanz, Germany; 4 Center for Critical Computational Studies, Goethe University Frankfurt, Berlin, Germany

**Keywords:** digital self-efficacy, digitalization-related knowledge, ICT-response beliefs, motivational regulation, technological pedagogical content knowledge, technological pedagogical knowledge, technology acceptance and competence beliefs

## Abstract

**Introduction:**

Digitalization is transforming education and increasing the demand for teachers’ competencies in integrating digital technologies into instruction. In this context, professional development plays a central role, yet it remains unclear how participation in digitalization-related professional development is associated with different dimensions of teachers’ professional competence and whether these relationships differ between STEM and non-STEM teachers.

**Methods:**

This study examines how participation in digitalization-related professional development is related to teachers’ motivational regulation, digital self-efficacy, digitalization-related knowledge (technological pedagogical knowledge and technological pedagogical content knowledge; TPK/TPACK), ICT-response beliefs, and technology acceptance and competence beliefs, and whether teaching a STEM subject moderates these relationships. A total of 1,031 teachers from North Rhine-Westphalia, Germany, completed an online survey.

**Results and discussion:**

The results indicate that participation in professional development—particularly when focused on digitalization—is associated with higher levels of motivational regulation, digital self-efficacy, and digitalization-related knowledge (TPACK). Differences between STEM and non-STEM teachers were generally small, although teachers without STEM subjects reported slightly lower TPACK. Correlational analyses revealed positive relationships among digital self-efficacy, digitalization-related knowledge, and technology-related beliefs.

## 1 Introduction

Digital technologies are increasingly integrated into classroom teaching and require teachers to adapt their instructional practices and professional roles. As a result, the integration of digitalization into teacher education and continuing professional development has become a central task for educational systems. International evidence shows that teachers frequently report a need for stronger support in digital pedagogy, particularly regarding how technology can be used to enhance student learning (OECD, 2019). Although many teachers participate in ICT-related professional development, participation rates and the time invested vary considerably across Europe (European Commission, 2020). These findings highlight the importance of professional development for supporting teachers’ professional competence in digital contexts. To design digital learning environments effectively, teachers need knowledge-based competencies that enable them to connect technological, pedagogical, and content knowledge in their instructional decisions. The TPACK framework (Technological Pedagogical Content Knowledge; Mishra and Koehler, 2006) provides a widely used reference framework for research on teaching with digital technologies. Given these developments, research on teacher professionalization increasingly emphasizes not only the acquisition of knowledge-based competencies, but also the role of teachers’ self-efficacy beliefs in shaping how such knowledge is enacted in instructional practice. Teachers’ confidence in their own ability to use digital tools meaningfully influences both their instructional behavior and their engagement in professional learning processes (Ertmer and Ottenbreit-Leftwich, 2010; Liu et al., 2025). Further relevant factors include teachers’ ICT-response beliefs and technology acceptance and competence beliefs, which reflect evaluative orientations toward the use of digital media in teaching (Davis et al., 1989; Scherer et al., 2019). Finally, motivational regulation is included as an indicator of teachers’ self-regulated engagement in professional development and learning activities (Ryan and Deci, 2000; Zhang and Liu, 2019). Together, these constructs provide an integrated framework for examining how participation in digitalization-related professional development is associated with teachers’ knowledge, beliefs, and motivational dispositions.

However, digitalization may not affect all school subjects to the same extent: While humanities focus on digitalization in terms of media skills and information work, STEM subjects, on the other hand, integrate digital technologies as a subject content element. In these fields, digital technologies like simulations, algorithmic tools, or sensor-based measurements are not merely instructional aids, but also central to how knowledge is produced and understood (Rutten et al., 2012). This suggests that teachers’ professional development in digital contexts may differ between STEM and non-STEM domains.

Overall, there is a high demand for professional development in the area of digitalization if schools are to respond appropriately to the challenges posed by the digital revolution. This is particularly relevant in Germany, as there are indications that the digital skills of teachers are rather average in an international comparison (Annemann et al., 2025).

Against this background, the present study adopts a focused analytical perspective on teachers’ professional competence in the context of digitalization. Rather than addressing a broad range of technology-related dispositions, the study concentrates on five core constructs that are consistently examined throughout the article: teachers’ motivational regulation, digital self-efficacy, digitalization-related knowledge (technological pedagogical knowledge and technological pedagogical content knowledge; TPK/TPACK), ICT-response beliefs, and technology acceptance and competence beliefs.

Participation in digitalization-related professional development is considered as a central explanatory factor for these constructs, while teaching a STEM subject is modeled as a potential moderating characteristic. Accordingly, the study investigates how professional development relates to these five dimensions of teachers’ professional competence and whether the relationships differ between STEM and non-STEM teachers.

## 2 Professional development for teachers

The (regular) participation in (formal) professional training is mandatory for teachers in Germany, and the participation rate is relatively high compared to international standards. However, there are hardly any binding requirements regarding the extent to which teachers in Germany must engage in professional development (Annemann et al., 2025; Richter and Richter, 2020; Röhl et al., 2023).

There have been numerous theoretical approaches aimed at modeling the prerequisites and determinants of effectiveness in teacher professional development. Within the German research discourse, one of the most influential frameworks is the adapted offer-and-use model of professional development by Lipowsky and Rzejak (2017). This model posits that the effectiveness of professional development is substantially shaped by a range of contextual variables, including the expertise of the facilitators, the scope, and quality of the training, as well as individual teacher characteristics such as prior knowledge, self-efficacy, motivation, interests, and beliefs. Similar conceptualizations to Lipowsky’s model can also be found in the international research literature, for example in the works of Desimone (2009) and Clarke and Hollingsworth (2002). Both models conceptualize professional learning as a process in which participation in professional development enhances teachers’ knowledge, beliefs, and attitudes, leading to changes in instructional practice and ultimately to improved student learning. Clarke and Hollingsworth’s (2002) framework, in particular, emphasizes that learning processes are mediated by individual teacher characteristics—such as prior knowledge, beliefs, and motivation—that interact dynamically with external professional learning opportunities within an interconnected system of personal, practical, and external domains. In addition to the individual development of specific teacher competencies, professional development programs also serve broader goals related to school and curriculum development (Aldorf, 2016; Borko, 2004), such as engaging with and developing competencies for working with digital media.

### 2.1 Participation in professional development

According to the theoretical approaches, requirements for participation in professional development include, among other things, teachers’ motivation and their prior knowledge (Clarke and Hollingsworth, 2002; Lipowsky and Rzejak, 2017). These aspects are particularly relevant in the present study and will be examined more closely. When it comes to willingness and requirements for participation in professional development, various aspects have been empirically studied – for example, in relation to years of teaching experience (Hauk et al., 2022), perceived practical relevance (Richter et al., 2019), satisfaction with previous training quality (Richter et al., 2018), or specific interest in the topic (Richter et al., 2019). Other motivating factors include career benefits (Cramer et al., 2019), the desire for social interaction, or personal development, with the latter two being positively correlated with beliefs in the effectiveness of training and perceived benefits (Hauk et al., 2022; Rzejak et al., 2014). Additionally, factors beyond personal disposition, such as travel time to the training location, the role of the school staff, and information availability, can also play a role (Richter and Vigerske, 2011).

Beyond individual interests and motivational factors related to professional development participation and topic selection, broader connections can be found in relation to school-type-specific requirements. For instance, Rotermund et al. (2017) found that primary and middle school teachers were more likely to participate in training on topics such as English as a Second Language or classroom management, whereas there were no significant differences in participation in digitalization-related topics compared to high school teachers. Some studies also indicate a slight trend that older teachers more frequently select training programs related to digitalization (Annemann et al., 2025; Rotermund et al., 2017). International comparisons show that, particularly in Germany, there are deficiencies in the participation in digitalization-related professional development and that German teachers tend to rate their digital competencies lower (Annemann et al., 2025).

### 2.2 Requirements and effects of professional development

Regarding the effectiveness of professional development, both international and German-speaking research have identified a number of overarching aspects that contribute to successful or effective training. However, the question of what constitutes a “successful” training is complex and depends on the criteria used – such as short- or long-term learning outcomes, changes in behavioral routines, or satisfaction with the training (Fussangel et al., 2016). It is generally assumed that the benefits of time-limited or “one-shot” professional development events are relatively low (Garet et al., 2001; Lipowsky and Rzejak, 2017), and the long-term effectiveness of teacher training is also questionable and insufficiently studied (Kraft et al., 2018; Wolf and Peele, 2019). Furthermore, professional development is associated with teachers’ motivational regulation and self-efficacy (DePiper et al., 2021; Liu et al., 2025; Trautner et al., 2025).

Motivational regulation refers to individuals’ deliberate efforts to manage their motivation in order to engage with and complete learning activities and is defined as “activities through which individuals purposefully act to initiate, maintain, or supplement their willingness to start, to provide work toward, or to complete a particular activity or goal” (Wolters, 2003, p. 191). Theoretically, motivational regulation is grounded in self-determination theory, which emphasizes processes of internalization and integration that support self-regulated forms of motivation (Ryan and Deci, 2000; Zhang and Liu, 2019), and in models of self-regulated learning that conceptualize motivational regulation as a dynamic process involving motivational monitoring and strategic control (Boekaerts, 1997; Kwok et al., 2022; Pintrich, 2004). Empirical research shows that motivational regulation is positively associated with learning engagement and performance across various learning environments (Mekler et al., 2017; Zhang and Liu, 2019). In professional learning contexts, motivational regulation has been found to depend on motivational beliefs, particularly the perceived professional relevance of learning activities, which promotes active motivation regulation and sustained learning engagement (Erol and Kurt, 2017; Zhang and Liu, 2019).

The concept of self-efficacy refers to an individual’s belief in their ability to cope with various challenges in everyday life and in professional contexts (see section 3.4; Bandura, 1997). In addition to mastery experiences, both accumulated experience and the acquisition of competencies can positively influence a person’s self-efficacy beliefs (Schüle et al., 2017). From the perspective of the offer-and-use model (Lipowsky and Rzejak, 2017), however, (digital) self-efficacy expectations should more appropriately be understood as a predictor of teachers’ engagement and learning within professional development contexts. A similar reasoning holds for the relationship between training and job satisfaction, which tends to be positive (Yoon and Kim, 2022). Looking at potential changes following digitalization-focused training, it becomes evident that in addition to increased digital self-efficacy, participation in digital or digitalization-focused training also positively impacts the integration of media in the classroom (Bremer and Antony, 2017; Gerick et al., 2017; Pan and Franklin, 2011; Yurkofsky et al., 2019).

## 3 Digitalization in schools

Digitalization represents a comprehensive and challenging transformation process within the education system, affecting both technological infrastructure and pedagogical-didactic development processes equally (Håkansson Lindqvist, 2019; Ilomäki and Lakkala, 2018; Timotheou et al., 2023). At the infrastructural or school level, digitalization offers opportunities to make internal school administrative and communication processes more efficient through learning management systems (Rott and Marouane, 2018). However, the state of digital infrastructure varies significantly, both between and within countries. Schools in disadvantaged or rural areas tend to be less well-equipped (OECD, 2023; UNESCO).

In terms of teaching, digital media can provide new opportunities for personalized learning, collaboration (both within and beyond school), as well as for learning motivation and engagement (e.g., through gamification) (Gan et al., 2015; Lin et al., 2017; Xie et al., 2019). In STEM education in particular, the potential of new digital applications is discussed with respect to increasing students’ interest in STEM subjects (Khalid et al., 2025; Wang et al., 2022). However, it is important to consider that these possibilities are not automatically realized by the mere availability of digital tools. Their effectiveness depends on many different factors, including their pedagogical use (Zierer, 2020). In this context, the role of the teacher and their individual (digital) competencies, attitudes, and interests is particularly relevant.

### 3.1 Teachers’ digital competencies

Various terms (e.g., digital competencies, digital literacy, media literacy), definitions, and frameworks exist to describe digital competence, each emphasizing different aspects. A widely used definition is provided by Hobbs (2010), who describes “digital and media literacy” as the “full range of cognitive, emotional and social competencies that includes the use of texts, tools and technologies; the skills of critical thinking and analysis; the practice of message composition and creativity; the ability to engage in reflection and ethical thinking; as well as active participation through teamwork and collaboration” (p. 17).

In teacher education research, digital competencies can be conceptualized within broader frameworks of professional competence. According to Baumert and Kunter (2006), teachers’ professional competence comprises professional knowledge, beliefs and values, motivational orientations, and self-regulation. While this model is not specific to digital contexts, its components provide a useful structure for understanding that technology integration depends not only on knowledge but also on teachers’ beliefs and motivational readiness.

Another established framework regarding teacher competencies is the TPACK model, which focuses primarily on the didactic use of media (Harris and Hofer, 2011). It distinguishes between pedagogical content knowledge (knowledge of teaching methods for specific content), technological pedagogical knowledge (knowledge of how technology supports learning processes), and technological content knowledge (knowledge of how subject content can be represented, conveyed, and worked on using digital technologies) (see Mishra and Koehler, 2006; Shulman, 1986). The TPACK model also forms the basis for measurement instruments used and refined in numerous studies (Schmidt et al., 2009; Voogt et al., 2013). In the present study, the TPACK model is used to assess teachers’ digitalization-related knowledge (competencies), as it provides an established framework for capturing pedagogical, technological, and content-related dimensions of technology-integrated teaching.

In STEM education, subject-specific requirements gain particular importance. Digitalization affects not only instructional tools but also the methods and approaches of scientific inquiry. The DiKoLAN framework (e.g., von Kotzebue et al., 2021) specifies science-specific digital competence areas such as data acquisition, data processing, and simulation/modelling, and explicitly relates them to the components of TPACK. This highlights that scientific knowledge production in digitalized laboratory and field contexts requires the integration of technological, pedagogical, and subject-specific understanding. Related operationalizations such as MaSter-Bio (Mahler and Arnold, 2022) assess prospective biology teachers’ academic self-concept regarding technological pedagogical content knowledge (TPACK), thereby indicating the need to consider subject-specific refinement for biology teaching beyond general technology skills.

Empirical research generally shows differences in self-assessed digital competencies by age, gender, and teaching subject. Female teachers tend to rate their competencies lower (Grande-de-Prado et al., 2020; Jiménez-Hernández et al., 2020; Palacios Rodríguez et al., 2023), as do older teachers (Jiménez-Hernández et al., 2020; Palacios Rodríguez et al., 2023; Zhao et al., 2021). In contrast, STEM teachers report significantly higher self-assessments of digital competence compared to teachers of other subjects (Ghomi and Redecker, 2019), although differences exist within STEM disciplines as well (Vieira et al., 2023).

### 3.2 Digital self-efficacy

Building on the constructs of digital competence and attitudes toward ICT integration discussed above, teachers’ digital self-efficacy represents a key requirement for successful teaching in the digital context (Peng et al., 2024; Ulfert-Blank and Schmidt, 2022). Digital self-efficacy refers to teachers’ confidence in their own ability to use digital technologies meaningfully and successfully in the planning, implementation, and reflection of technology-supported teaching and learning processes (Garzon and Garzon, 2023). Rooted in social cognitive theory, Bandura (1997) defined self-efficacy beliefs as “beliefs in one’s capabilities to organize and execute the courses of action required to produce given attainments” (p. 3).

Teachers’ self-efficacy refers to teachers’ confidence in their own ability to plan, implement, and reflect on teaching and learning processes in order to achieve specific educational goals (Yang and Du, 2024). Teachers with high self-efficacy exhibit greater job satisfaction, engagement, professional commitment, and resilience to stress (Aloe et al., 2014; Bach, 2022; Lazarides and Warner, 2020; Zee and Koomen, 2016) and are less susceptible to burnout (Aloe et al., 2014; Daniilidou et al., 2020; Zee and Koomen, 2016). Building on the general understanding of self-efficacy, digital self-efficacy is defined as teachers’ confidence in their own abilities to use digital technologies meaningfully and successfully during the planning, implementation, and reflection of technology-supported teaching and learning processes (Garzon and Garzon, 2023; Yang and Du, 2024).

Empirically, digital self-efficacy has been linked to several aspects of teachers’ instructional practice. Empirical findings suggest that the frequency of media use in the classroom correlates with teachers’ digital self-efficacy expectations (Hatlevik and Hatlevik, 2018; Li et al., 2018; Paetsch et al., 2023; Peng et al., 2024). Moreover, a positive relationship exists between digital self-efficacy and teachers’ digital competencies (Paetsch et al., 2023; Peng et al., 2024; Ulfert-Blank and Schmidt, 2022; Wang and Chu, 2023; Zeng et al., 2022). Moreover, Zeng et al. (2022) conducted a meta-analysis which found a moderate positive correlation between teachers’ information technology integration self-efficacy and TPACK. While constructs such as TPACK primarily reflect teachers’ perceived knowledge regarding the integration of digital tools, digital self-efficacy emphasizes the belief in one’s capability to apply such knowledge effectively in practice. Thus, self-efficacy serves as a motivational bridge between competence and behavior.

Digital self-efficacy plays a crucial role in the professional development of STEM teachers, as it significantly influences their willingness and ability to integrate technology into teaching and to design effective, technology-enhanced learning environments (Ertmer and Ottenbreit-Leftwich, 2010; Liu et al., 2025). However, teachers often lack professional knowledge of digital technologies and design thinking, as these are rarely part of their initial training (Eriksson et al., 2018). Accordingly, studies highlight the importance of professional development aimed at strengthening digital competencies in order to enhance teachers’ self-efficacy in digital teaching and improve curriculum quality (Garzon and Garzon, 2023; Sehar and Alwi, 2023; Shi et al., 2025). Liu et al. (2025) further demonstrate in their meta-analysis that professional development has a moderately positive effect on the self-efficacy of STEM teachers. Teachers participating in targeted and practice-oriented training report higher levels of digital and technological self-efficacy, especially when programs are hands-on and application-based. Longer and more intensive training phases produce stronger effects, whereas very large group formats tend to be less effective. Non-traditional approaches such as workshops or communities of practice have proven particularly successful, as they foster collaborative learning and immediate application. Overall, the literature emphasizes that continuous, practice-based professional development is crucial for strengthening STEM teachers’ digital self-efficacy and enabling them to integrate technology effectively into their teaching practice.

As in other areas of self-efficacy (Martin et al., 2008; Tschannen-Moran and McMaster, 2009; Zhang et al., 2021), digital self-efficacy expectations are also related to participation in media-related professional development (Drossel and Eickelmann, 2017; Kao et al., 2011), although the direction of the effect is not always clear – self-efficacy is modeled both as a predictor and as a dependent variable. Professional development that targets both digital competencies and self-efficacy can therefore enhance teachers’ confidence to implement technology-supported instruction. And given the established link between self-efficacy and teachers’ willingness to engage in professional development, digital self-efficacy is assumed to play a key role in shaping participation patterns and perceived outcomes of media-related training.

### 3.3 ICT-response beliefs and technology acceptance

Compared to digital competence and self-efficacy, other areas such as interest in ICT (Information and Communication Technologies) are less researched. ICT interest is conceptualized as a relatively enduring predisposition to engage with ICT-related topics, tasks, or activities, typically accompanied by positive affect and value-related evaluations (Goldhammer et al., 2016; Krapp, 2005). Examining ICT interest is relevant because it is closely linked to learning motivation, and higher levels of interest can foster the development of competencies in the respective domain (Eccles and Wigfield, 2002; Krapp, 2005). Empirical studies have shown positive, though mostly modest, correlations between ICT interest and media-related competencies (Christoph et al., 2015; Fraillon et al., 2014; Juhaňák et al., 2019; Rubach and Lazarides, 2019). Only limited findings exist for teachers, suggesting that (prospective) female teachers report lower ICT interest (Besa et al., 2021; Fraillon et al., 2014).

Beyond general interest, teachers’ responses to the instructional use of digital media can be described as ICT-response beliefs, referring to their evaluations of the pedagogical usefulness of digital technologies in classroom practice. Such beliefs are associated with teachers’ actual use of digital media and their willingness to integrate technology into teaching (Ertmer and Ottenbreit-Leftwich, 2010; Scherer et al., 2019), and more positive beliefs correspond to more frequent and pedagogically meaningful technology integration (Petko, 2012).

While ICT interest reflects a motivational disposition and ICT-response beliefs refer to instructional evaluations, technology acceptance addresses more general beliefs about the usefulness and usability of digital technologies. According to general attitude–behavior models, such evaluations influence intentions and, consequently, behavior (Fishbein and Ajzen, 1975). In technology-related contexts, these beliefs are commonly specified within the Technology Acceptance Model (TAM; Davis et al., 1989), which identifies perceived usefulness and perceived ease of use as key determinants of technology use and has been widely applied in educational research to explain teachers’ use of digital media (Mailizar et al., 2021; Weng et al., 2018; Wong et al., 2012).

Empirical studies examining teachers’ integration provide mixed evidence regarding the role of sociodemographic characteristics. Studies generally show no significant gender differences in technology acceptance among teachers (Cavas et al., 2009; Islahi and Nasrin, 2019; Pamuk and Peker, 2009; Semerci, 2018). In studies involving other population groups, men tend to show slightly more positive attitudes, though the effect sizes are small (Cai et al., 2017; Siddiq and Scherer, 2019). Regarding age, Cavas et al. (2009) found that teachers aged 20–35 had significantly more positive attitudes toward ICT integration than those in the 36–49 or 50 + age groups. Studies examining moderating effects of age or professional status (pre-service vs. in-service teachers) on TAM variables reported no to small effects (Martín-García et al., 2019; Sánchez-Mena et al., 2017; Scherer et al., 2019). As for differences based on subject area, no empirically confirmed statements can be made. Barak (2014) compared attitudes toward ICT integration in two cohorts of STEM teachers (2006 vs. 2012) without comparing them to other subject groups.

In the present study, these evaluative orientations toward digital technologies are differentiated into two related but distinct constructs: ICT-response beliefs, referring to teachers’ evaluations of the pedagogical usefulness of digital media in classroom practice, and technology acceptance and competence beliefs, reflecting more general beliefs about the usefulness of digital technologies and one’s capability to work with them.

## 4 Aim and research questions

In summary, knowledge-based competencies (TPK / TPACK), and digital self-efficacy appear to be relevant and interlinked factors influencing teachers’ ICT-response beliefs. Based on the theoretical considerations outlined above, participation in digitalization-related professional development is examined in relation to teachers’ motivational regulation, digital self-efficacy, digitalization-related knowledge (TPK/TPACK), ICT-response beliefs, and technology acceptance and competence beliefs. Teaching a STEM subject is considered as a potential moderating characteristic when comparing teachers across these dimensions. Among teachers in Germany, these competencies and self-efficacy beliefs still seem to offer room for improvement. This is particularly evident in STEM education, where media play a multifaceted role (Becker et al., 2020; Boeve-De Pauw et al., 2024). Professional development programs for teachers can contribute meaningfully in this regard (Fernández Batanero et al., 2020; Liu et al., 2025)—provided, however, that they are attended especially by those individuals who exhibit the greatest need for development. However, there is a lack of empirical studies investigating whether and how STEM-specific digitalization-related professional development affects teachers’ TPACK competencies and their digital self-efficacy. Moreover, most studies focusing on STEM teachers either rely on small sample sizes or do not specifically examine professional development and its outcomes. Accordingly, the present article seeks to address the following research questions:

RQ1: To what extent do teachers with and without a STEM subject differ with regard to

- motivational regulation,
- digitalization-related knowledge (TPK/TPACK),
- digital self-efficacy,
- ICT-response beliefs, and
- technology acceptance and competence beliefs?

RQ2: To what extent do teachers who participate in digitalization-related professional development, teachers who participate in professional development without a digitalization focus, and teachers who do not participate in professional development differ with regard to

- demographic and professional characteristics (age, gender, professional experience, school type, subject taught),
- motivational regulation,
- digitalization-related knowledge (TPK/TPACK)
- digital self-efficacy,
- ICT-response beliefs, and
- technology acceptance and competence beliefs?

RQ3: How are teachers’ motivational regulation, digital self-efficacy, digitalization-related knowledge (TPK/TPACK), ICT-response beliefs, and technology acceptance and competence beliefs related to each other and to teachers’ age and professional experience?

## 5 Methods and study design

### 5.1 Sample

A total of *N* = 1031 (prospective) teachers from the German state of North Rhine-Westphalia participated in the current study. On average, they were 42 years old (*SD* = 11.46 years; age_min_ = 22 years, age_max_ = 65 years). 68.7% of the teachers were female. While 988 teachers (95.8%) stated that they had completed their traineeship, 14 (1.4%) were currently completing their traineeship or had not completed it. A total of 32 teachers (3.1%) reported a lateral or side entry. The average professional experience of the teachers surveyed was around 16 years (*SD* = 11.60 years; professional experience_min_ = 0 years, professional experience_max_ = 41 years). A summary of the sample composition can be found in Table 1.

**Table 1:** Summary of the sample composition.

***N***	**Age(*M ± SD*) [years]**	**Gender(f/m/no gender) [%]**	**Professional experience(*M ± SD*) [years]**	**Lateral and side entryYes/No [%]**
1,031	42.08 ± 11.46	68.7/31.2/0.1	16.15 ± 11.60	3.1/96.9

The majority of teachers teach at an elementary school (37.9%). In addition, large numbers of teachers work at higher track secondary schools (“Gymnasium” = 24.3%, “Gesamtschule” = 18.6%). 16% teach at lower secondary schools (“Realschule” = 4.8%, ‘Hauptschule’ = 8.1%, “Sekundarschule” = 3.1%). A further 2.6% of teachers work at a vocational college.

While 479 teachers did not study a STEM subject (46.5%) and 485 teachers studied one STEM subject (47.0%), 62 teachers stated that they studied two STEM subjects (6.0%). Three and two teachers surveyed reported studying three and four STEM subjects, respectively. For the following analyses, the teachers are differentiated into the groups “No STEM subject”, “One STEM subject” and “Min. two STEM subjects”. For further analysis steps, the teachers were also asked about their participation in various professional development programs within the last 2 years. According to their answers, the teachers were divided into the groups “Professional development related to digitalization” (*n* = 777; 78.7%), “Professional development not related to digitalization” (*n* = 132; 13.4%) and “No Professional development” (*n* = 78; 7.9%). This study was conducted using a cross-sectional study design. At one measurement point in 2024, a questionnaire study was conducted in online format with teachers from the region of East Westphalia (North Rhine-Westphalia) in Germany. The teachers were recruited for participation via the Bielefeld school network, and the survey was conducted using the survey software SoSci Survey.

### 5.2 Test instruments

A short scale on general self-efficacy (Beierlein et al., 2012) adapted to the use of digital media was applied to measure the digital self-efficacy of the participating teachers. This scale also comprised three items. Two subscales of the questionnaire by Stinken-Rösner (2021) and Stinken-Rösner et al. (2023) were used to measure the teachers’ digitalization-related knowledge. Teachers’ technological and pedagogical knowledge (TPK) was assessed using four items, while five items were included to record technological and pedagogical content knowledge (TPACK). In addition, the teachers’ ICT-response beliefs were measured using seven items from the Schmidt and Reintjes (2020) scale. To check the factor structure of these four scales, a principal component analysis was performed. The factor analysis was conducted exclusively with the four scales on Digital Self-Efficacy, TPACK, TPK, and ICT-Response Beliefs, which can be justified from the perspective of construct validity. Only these four scales were examined using factor analysis because, both theoretically and based on an item analysis, they represent the same (or closely related) constructs, and their internal structure must be examined to ensure construct validity. While the four analyzed scales focus on competence beliefs and professional knowledge in the context of digital teaching, motivational regulation refers to learning- or work-related self-regulation processes, and technology acceptance concerns attitudes and behavioral intentions toward technologies. Although these constructs are theoretically related, they do not belong to the same latent structure. Therefore, they were not included in the factor analysis. The Kaiser-Meyer-Olkin (KMO) measure of sampling adequacy was 0.79, and Bartlett’s test of Sphericity was significant (*p* < 0.001). Solely factors with an eigenvalue ≥ 1 were included. The exploratory factor analysis indicated a four-factor structure in line with theory, explaining 65.20% of the total variance (Table 2).

**Table 2:** Exploratory factor analysis of the study instruments.

Item	**Factor 1:** ICT-response beliefs	**Factor 2:** TPACK	**Factor 3:** Digital self-efficacy	**Factor 4:** TPK
*I prefer to teach without digital media.*	0.85			
*New technologies are changing teaching for the better.*	0.82			
*New technologies make teaching more interesting.*	0.81			
*Digital media are a useful addition to my lessons.*	0.78			
*Digital media enrich my teaching.*	0.77			
*The use of digital media takes up unnecessary teaching time.*	0.71			
*The use of digital media in teaching is necessary in today’s world.*	0.70			
*I can design problem-centered learning environments that support students in applying and deepening subject content with the help of digital media (e.g., simulations, online materials, etc.).*		0.86		
*I can design learning environments that enable students to learn subject content independently with the help of digital media (e.g., blogs, webquests, etc.).*		0.86		
*I can design student-centered learning environments in which subject content, pedagogical principles, and digital media are appropriately integrated.*		0.85		
*I can formulate challenging discussion topics on subject content and support online collaboration among students using appropriate digital media (e.g., Google Docs, forums, etc.).*		0.82		
*I can design learning environments that support students in presenting subject content in various ways using digital media (e.g., mind maps, wikis, etc.).*		0.80		
*I can easily overcome most problems with using digital media in my subject on my own.*			0.90	
*I can rely on my skills in difficult situations when using digital media in my subject.*			0.86	
*I can usually cope well with even strenuous and complicated challenges involving the use of digital media in my subject.*			0.85	
*I can support students in presenting content in different ways using digital media (e.g., texts, graphics, formulas, etc.).*				0.78
*I can support students in planning and monitoring their own learning process with digital media.*				0.75
*I can use digital media to give my students everyday examples.*				0.72
*I can support students in cooperating with each other with the help of digital media.*				0.67
eigenvalue	4.81	3.47	2.19	1.93
proportion of variance [%]	25.29	18.24	11.51	10.15
internal consistency (Cronbach’s Alpha)	0.89	0.90	0.84	0.72
selectivity of items	0.60–0.78	0.72–0.79	0.67–0.76	0.45–0.57

Factor loadings from |0.52| are reported. KMO = 0.79. 65.20% of the variance.

The teachers’ motivational regulation with regard to teaching was assessed using three items of the Intrinsic Regulation subscale from the Motivational Regulation in Learning Scale (SMR-L, Thomas and Müller, 2016), adapted to the context. The short scale for measuring technology readiness (Neyer et al., 2012) was used to assess teachers’ technology acceptance and technology competence beliefs.

All items/scales were measured using a five-point rating scale (1 = strongly disagree to 5 = strongly agree). The internal consistencies, measured using Cronbach’s alpha, were in a satisfactory range for all (sub)scales (Taber, 2018; Table 3).

**Table 3:** Example items, item numbers, internal consistencies for the investigated variables.

**Variable**	**Example item**	**Item number**	**Cronbach’s Alpha**
Motivational regulation	*I really enjoy teaching and working in my profession.*	3	0.68
Technology acceptance and competence beliefs	*I am interested in using the latest technical equipment.*	6	0.91

### 5.3 Statistical analyses

Several univariate and multivariate analyses of variance (ANOVA, MANOVA) were conducted to compare teachers with and without a STEM subject in terms of their motivational regulation, digital self-efficacy, digitalization-related knowledge, ICT-response beliefs, technology acceptance and technology competence beliefs in the classroom (research question 1). ANOVAs and MANOVAs were also used to compare teachers who participated in different training formats with and without a focus on digitalization and teachers who did not participate in training courses with regard to the variables mentioned (research question 2).

In addition to testing for normal distribution of the variables using Shapiro–Wilk tests, Levene tests were carried out to test variance homogeneity. These requirements were found to be fulfilled. Subsequent Bonferroni-corrected posthoc tests were used to examine differences between the described groups of teachers with regard to their subject composition and participation in professional development. Chi-square procedures were used to examine the distribution of these teachers among the different groups described in terms of their gender, school type and subjects taught (research question 2).

Bivariate correlation analyses were performed to investigate the relationships between the variables examined (motivational regulation, digital self-efficacy, digitalization-related knowledge and ICT-response beliefs, technology acceptance and competence beliefs, age, professional experience).

## 6 Results

### 6.1 Research question 1: differences between teachers with and without a STEM subject

The univariate analyses of variance revealed no significant differences between teachers with different numbers of STEM subjects in terms of motivational regulation and digital self-efficacy. Teachers in all groups reported a rather high level of motivational regulation in relation to teaching and a high level of digital self-efficacy.

With regard to digitalization-related knowledge, no significant differences were found for the teachers’ technological-pedagogical knowledge. Significant differences with small effect sizes were evident for the teachers’ technological-pedagogical content knowledge (TPACK) (question 1b). The subsequent Bonferroni-corrected posthoc tests showed that teachers who do not teach a STEM subject differ significantly from those who teach a STEM subject (*p* < 0.05, M_diff_ = 0.16, 95%-CI[0.00, 0.31]).

No significant differences were found in the comparison of teachers with regard to their ICT-response beliefs and their technology acceptance and competence beliefs (see Table 4).

**Table 4:** Mean values, standard deviation (differentiated by teachers with different subject compositions), between-subjects effects for the investigated variables.

**Variable**	**Subject composition**	***M***	***SD***	**Between-subject effects**
Motivational regulation	Without STEM subject	3.42	0.88	*F*(2,1,027) = 0.02, *p* = ns
	One STEM subject	3.41	0.94	
	Min. two STEM subjects	3.43	0.95	
TPK	Without STEM subject	3.52	0.84	*F*(2,1,014) = 1.73, *p* = ns
	One STEM subject	3.44	0.88	
	Min. two STEM subjects	3.62	0.80	
TPACK	Without STEM subject	3.04	0.99	*F*(2,1,014) = 3.08, *p* < 0.05, η^2^ = 0.01
	One STEM subject	3.20	1.01	
	Min. two STEM subjects	3.19	1.00	
Digital self-efficacy	Without STEM subject	3.13	0.99	*F*(2,1,010) = 1.04, *p* = ns
	One STEM subject	3.20	1.07	
	Min. two STEM subjects	3.30	1.04	
ICT-response beliefs	Without STEM subject	3.48	0.87	*F*(2,1,012) = 0.62, *p* = ns
	One STEM subject	3.54	0.94	
	Min. two STEM subjects	3.55	0.95	
Technology acceptance and competence beliefs	Without STEM subject	3.19	1.03	*F*(2,1,012) = 0.85, *p* = ns
	One STEM subject	3.20	1.01	
	Min. two STEM subjects	3.36	1.06	

The results show that, for the most part, there are no significant differences between teachers with and without STEM subjects in terms of their digitalization-related skills and beliefs. Only with respect to their self-assessed TPACK do teachers without STEM subjects report a significantly lower level.

### 6.2 Research question 2: differences between teachers in different training formats

With regard to age and professional experience (question 2a), the univariate analyses of variance showed significant differences between the teachers who took part in different training formats or did not attend any training [age: *F*(2,983) = 10.57, *p* < 0.001, η^2^ = 0.02; professional experience: *F*(2,982) = 9.50, *p* < 0.001, η^2^ = 0.02]. The subsequent posthoc tests showed that the teachers who took part in professional development related to digitalization were significantly younger than the teachers who took part in professional development not related to digitalization (*p* < 0.001, *M*_diff_ = 0.16, 95%-CI[0.00, 0.31]) and than the teachers who did not take part in professional development (*p* < 0.05, *M*_diff_ = 0.16, 95%-CI[0.00, 0.31]). In terms of professional experience, the posthoc test only revealed a significant difference between teachers who attended training with and without a digital focus. Teachers who took part in training with a focus on digitalization reported significantly less professional experience in comparison (*p* < 0.001, M_diff_ = 0.16, 95%-CI[0.00, 0.31]). Regarding teachers’ gender, school type, and subject taught the chi-square-tests revealed significant effects [gender: χ^2^(6,943) = 13.92, *p* < 0.05; school type: χ^2^(12,983) = 495,04, *p* < 0.001; subjects: χ^2^(4,987) = 10.71, *p* < 0.05]. The gender distribution across the groups with regard to training participation showed that the proportion of female teachers who took part in training courses related to digitalization was relatively higher than in the other training groups. Concerning the type of school, it can be seen that primary school teachers took part in training courses related to digitalization relatively frequently compared to the other training groups, while teachers at lower secondary schools attended a relatively high number of training courses without a focus on digitalization. The chi-square tests for the distribution of teachers with different subjects showed that teachers with a STEM subject took part in training courses with a focus on digitalization relatively frequently comparing the training groups.

The univariate analysis of variance also revealed significant differences between the groups of teachers described for motivational regulation [*F*(2,983) = 12.03, *p* < 0.001, η^2^ = 0.02] (question 2b). Here, the posthoc analyses showed that the teachers who participated in professional development with and without reference to digitalization reported significantly higher motivational regulation than the teachers who did not participate in professional development (*p* < 0.001, *M*_diff_ = 0.52, 95%-CI[0.27, 0.78]; *p* < 0.001, *M*_diff_ = 0.53, 95%-CI[0.22, 0.84]). With regard to digitalization-related knowledge (question 2c), the multivariate analysis of variance did not reveal any significant differences for the TPK, while the teachers differed significantly with regard to their TPACK [*F*(2,972) = 28.68, *p* < 0.001, η^2^ = 0.06]. The following *post-hoc* analyses showed that the teachers who took part in training courses without reference to digitalization differed significantly from both the teachers who took part in training courses with reference to digitalization and those who did not take part in training courses (*p* < 0.001, *M*_diff_ = 0.40, 95%-CI[0.17, 0.62]; *p* < 0.01, *M*_diff_ = 0.40, 95%-CI[0.06, 0.74]). Teachers who took part in digitalization training also differed from those who did not (*p* < 0.001, *M*_diff_ = 0.79, 95%-CI[0.51, 1.07]). The latter reported the lowest TPACK. In addition, significant differences were found with regard to the teachers’ digital self-efficacy (question 2d) [*F*(2,969) = 7.83, *p* < 0.001, η^2^ = 0.02]. According to the posthoc tests, these findings indicate significant differences in the comparison of teachers who participated in training courses with and without a focus on digitalization (*p* < 0.001, *M*_diff_ = 0.37, 95%-CI[0.13, 0.60]). Teachers who attended training courses without reference to digital media reported the lowest digital self-efficacy.

No significant differences were found between the groups of teachers described for their ICT-response beliefs [*F*(2,978) = 2.59, *p* = ns] or for their acceptance of technology and conviction in technological competence for teaching [*F*(2,978) = 1.80, *p* = ns] (question 2e and f; Table 5).

**Table 5:** Mean values, standard deviation (differentiated according to different professional development formats), between-subjects effects for the investigated variables.

**Variable**	**Professional development format**	****M****	***SD***	**Between-subject effects**
Age	PDwD	41.23	11.58	*F*(2,983) = 10.57, *p* < 0.001, η^2^ = 0.02
	PDwoD	45.56	9.54	
	No PD	44.81	12.12	
Professional experience	PDwD	15.34	11.72	*F*(2,982) = 9.50, *p* < 0.001, η^2^ = 0.02
	PDwoD	19.70	9.66	
	No PD	18.28	12.67	
Motivational regulation	PDwD	3.45	0.88	*F*(2,983) = 12.03, *p* < 0.001, η^2^ = 0.02
	PDwoD	3.46	1.05	
	No PD	2.93	0.90	
TPK	PDwD	3.47	0.86	*F*(2,972) = 1.69, *p* = ns
	PDwoD	3.55	0.83	
	No PD	3.65	0.80	
TPACK	PDwD	3.24	1.03	*F*(2,972) = 28.68, *p* < 0.001, η^2^ = 0.06
	PDwoD	2.85	0.80	
	No PD	2.45	0.73	
Digital self-efficacy	PDwD	3.24	1.03	*F*(2,969) = 7.83, *p* < 0.001, η^2^ = 0.02
	PDwoD	2.88	1.00	
	No PD	3.03	1.01	
ICT-response beliefs	PDwD	3.49	0.90	*F*(2,978) = 2.59, *p* = ns
	PDwoD	3.68	0.91	
	No PD	3.46	1.00	
Technology acceptance and competence beliefs	PDwD	3.18	1.03	*F*(2,978) = 1.80, *p* = ns
	PDwoD	3.33	1.02	
	No PD	3.33	0.94	

Professional development with focus on digitalization = PDwD; professional development without focus on digitalization = PDwoD; no professional development = No PD.

### 6.3 Research question 3: correlations between variables

The correlation analysis revealed several significant relationships among the key variables. Digital self-efficacy was positively and significantly associated with teachers’ reported TPK and TPACK scores, as well as with their ICT-related beliefs, technology acceptance, and competence beliefs. Furthermore, it was correlated with both teachers’ age and professional experience, indicating that these personal and career-related factors may play a role in shaping digital self-efficacy. Similarly, TPACK showed significant associations with ICT-response beliefs, technology acceptance, and competence beliefs, highlighting the interconnectedness of teachers’ technological knowledge, attitudes, and self-perceptions. ICT-response beliefs were significantly linked to teachers’ age and professional experience, suggesting that longer teaching experience and older age may influence how educators respond to ICT-related challenges. However, no significant correlations were found between motivational regulation and teachers’ ICT-related beliefs, indicating that motivational aspects may not directly influence these specific concerns. Overall, the results suggest that digital self-efficacy and TPACK are closely tied to a range of personal, professional, and attitudinal factors, while motivational regulation appears to operate independently of ICT-related worries. These findings are further illustrated in Table 6.

**Table 6:** Results of the bivariate correlation analysis.

Variable	***M***	***SD***	1	2	3	4	5	6	7
1 Motivational regulation	3.41	0.91							
2 Digital self-efficacy	3.18	1.03	0.12**						
3 TPK	3.49	0.86	0.01	0.09**					
4 TPACK	3.13	1.01	0.08**	0.13**	0.22**				
5 ICT-response beliefs	3.51	0.91	0.04	0.07*	0.16**	0.10**			
6 Technology acceptance and competence beliefs	3.20	1.03	0.10**	0.10**	0.07*	0.13**	0.002		
7 Age	42.08	11.46	−0.09**	−0.14**	−0.01	−0.18**	−0.07*	−0.05	
8 Professional experience	16.15	11.60	−0.10**	−0.14**	−0.01	−0.17**	−0.08**	−0.06	0.96**

* *p* < .05, ** *p* < .01.

## 7 Discussion and limitations

This study sought to examine differences across various domains of teachers’ self-assessed knowledge-based competencies and motivational variables in the context of professional development.

Concerning the first research question, the findings revealed—contrary to the findings of Ghomi and Redecker (2019)— no significant differences between teachers with and without STEM subjects in terms of motivational regulation, digital self-efficacy, technological-pedagogical knowledge (TPK), ICT-response beliefs, or technology acceptance and competence beliefs. The slightly lower TPACK values among non-STEM teachers can be interpreted in light of the TPACK framework (Mishra and Koehler, 2006), which emphasizes the integration of technological, pedagogical, and subject-specific knowledge. Because STEM subjects often involve simulation software, data analysis tools, or digital measurement technologies, opportunities to connect content and technology may be more readily available in these domains. The present findings therefore suggest that differences are less a matter of subject ability and more a matter of domain-specific opportunities to develop integrated knowledge structures. With regard to the second research question, the analyses revealed significant differences between teachers who participated in various types of professional development or did not attend any professional development in terms of age, professional experience, gender, school type, subject taught, motivational regulation, digital self-efficacy, and technological-pedagogical content knowledge (TPACK). Contrary to prior empirical findings (e.g., Rotermund et al., 2017), teachers engaging in digitalization-related professional development were significantly younger and less experienced than those attending non-digital professional development or no professional development at all.

Furthermore, the present data confirm expected relationships, such as higher self-assessed digitalization-related knowledge and digital self-efficacy beliefs among younger teachers (Zhao et al., 2021). Moreover, the analyses carried out in response to the third research question show significant correlations between the various scales addressing different aspects of digitalization (in schools) are also expected (Christoph et al., 2015; Fraillon et al., 2014), although the correlation coefficients are generally rather low. Likewise, the markedly lower motivational regulation observed among teachers who have not participated in professional development over the past 2 years is unsurprising.

These findings highlight the importance of considering demographic and professional factors when designing professional development programs. The higher engagement of younger, less experienced, female, and primary school teachers in digital training suggests potential gaps in reach to other groups, such as more experienced or male teachers. Against this background, these prerequisites should be considered when designing inclusive training formats tailored to the needs of teachers. The correlation analyses thus reveal significant correlations between self-assessed digitalization-related skills and digital self-efficacy. The association between professional development participation and higher digital self-efficacy aligns with social cognitive theory (Bandura, 1997), according to which mastery experiences and guided practice constitute central sources of self-efficacy beliefs. Professional development may therefore function not only as knowledge acquisition but also as a structured opportunity for successful technology use, thereby reinforcing teachers’ confidence in their instructional capabilities. Some limitations need to be acknowledged. First, all collected data consist of self-assessments. The extent to which these reflect actual competencies acquired through professional development remains debatable, as research on teacher competence measurement shows that self-assessment instruments may not capture all relevant dimensions or align sufficiently with external measures (Gehrmann et al., 2025), and that, in the context of online professional development, self-reported knowledge gains often fail to correlate with objectively assessed gains (Li and Copur-Gencturk, 2025). Although several group differences reached statistical significance, the observed effect sizes were generally small. The findings suggest that participation in digitalization-related professional development is associated with modest shifts across multiple competence-related dimensions instead of substantial changes in individual constructs.

The use of only three items to assess key constructs was a pragmatic decision to ensure efficient data collection and maintain response rates in a large-scale survey. Although the scales showed satisfactory reliability, the limited number of items represents a methodological limitation. Short scales may not fully capture the complexity of the constructs and can reduce measurement validity and sensitivity to subtle differences. Consequently, the findings should be interpreted cautiously. Future research should therefore employ more extensive and potentially multidimensional or context-specific instruments to improve measurement quality and allow for more detailed insights into underlying processes. The categorization of professional development into the broad groups “with digital focus,” “without digital focus,” and “none” simplified data analysis but limited insight into the actual nature of the training. This approach does not capture relevant dimensions such as intensity, duration, quality, or specific content. As a result, substantially different formats (e.g., a brief workshop versus an extended program with implementation phases) may be treated as equivalent, potentially obscuring relationships between teachers’ competencies and their participation in professional development. Future research should therefore collect more detailed information on characteristics such as duration, frequency, instructional design, and opportunities for hands-on practice to enable more differentiated analyses.

Furthermore, due to the cross-sectional design of the study, it remains unclear whether the observed correlations might also reflect a Matthew effect, as suggested by Richter et al. (2021) in relation to professional development, and well-documented in the context of digital inequality (DiMaggio and Hargittai, 2001; van Dijk, 2005), where those who already possess more resources and competencies tend to benefit more from access and training opportunities, thereby deepening existing disparities. They found connections between subject-specific knowledge and enthusiasm for the subject as relevant factors influencing (subject-specific) training participation. Concerns about a knowledge gap in the population due to differing levels of willingness to engage in further education have existed for some time (cf. Heuer, 2001), and such phenomena are known from other areas of adult education: Blossfeld et al. (2020) identified a Matthew effect in adult learning across countries, showing that particularly in non-formal learning settings, prior education is beneficial. Considering the desired outcomes of digital teacher professional development, both competencies and self-efficacy beliefs are key factors, especially here. Nevertheless, in the study at hand, it remains uncertain whether teachers participate in digitalization-related professional development because they already have a strong interest in digital topics or perceive themselves as particularly competent in this area, or conversely, whether such interest and perceived competence are enhanced by participating in such training. Still, from our perspective, it is worthwhile—based on the present findings—to reflect on the mechanisms that might be at work. It is particularly striking that the constructs measured show not only differences between individuals who participated in professional development programs with and without a digitalization focus, but also that teachers who did not participate in any professional development at all reported the highest levels of digital self-efficacy. On the other hand, this group rated themselves lowest in terms of TPACK. Interpreted in light of models of teacher professional growth (Borko, 2004; Clarke and Hollingsworth, 2002), professional learning opportunities may contribute to more differentiated knowledge structures and thereby to more cautious self-assessments, whereas teachers without such learning opportunities may rely more strongly on general impressions rather than on structured experiences.

When compared to existing research, these findings are largely consistent with the assumptions of Richter et al. (2019, 2021), who identified pre-existing topical interest as a relevant factor in the selection of professional development content. At the same time, however, the fact that older teachers are particularly likely to select professional development in the area of digitalization (see Rotermund et al., 2017) suggests that such conclusions should be interpreted with greater nuance—especially if one assumes correlations between age and digital competence or interest. Interestingly, our data do not support this relationship; rather, it was primarily younger teachers who reported participating in digitalization-related training.

The fact that our findings—both regarding this issue and the overall high level of participation in digitalization-related professional development—partially diverge from earlier research may be attributable to a historical disruption: the COVID-19 pandemic. Since our survey was conducted in 2024 and focused on professional development participation over the previous 2 years, the captured period coincides with changes in schools and teaching caused by the COVID-19 pandemic. The COVID-19 pandemic acted as a catalyst for the rapid digitalization of educational settings, highlighting significant gaps in teachers’ digital competencies and thereby intensifying the demand for targeted professional development (König et al., 2020). As a result, digitalization-related training programs for teachers gained increased relevance, both in addressing immediate challenges and in promoting long-term integration of digital tools in instruction.

Taken together, the findings suggest that the integration of digital technologies in schools should not primarily be understood as a question of subject domain or access to technical tools, but as a matter of professional learning opportunities. The comparatively small differences between STEM and non-STEM teachers indicate that digital teaching competence is not restricted to particular subjects but can be developed across domains. Professional development may therefore be more effective when it emphasizes the pedagogical integration of technology into subject-related learning processes rather than the introduction of isolated applications. From an educational perspective, this means that effective support for teachers is likely to require sustained and practice-oriented learning opportunities that connect technological tools with concrete instructional scenarios rather than short-term training on isolated software.

## Data Availability

The raw data supporting the conclusions of this article will be made available by the authors, without undue reservation.
